# Dermatology (Beau’s Lines) and Forensic Pathology (Harris Lines and Linear Enamel Hypoplasia) Signs of Growth Arrest: A Case Report and Literature Review

**DOI:** 10.7759/cureus.85190

**Published:** 2025-06-01

**Authors:** Philip R Cohen

**Affiliations:** 1 Dermatology, University of California Davis Medical Center, Sacramento, USA; 2 Dermatology, Touro University California College of Osteopathic Medicine, Vallejo, USA; 3 Maples Center for Forensic Medicine, University of Florida College of Medicine, Gainesville, USA

**Keywords:** beau’s line, dermatology, enamel, forensic, harris, hypoplasia, linear, nail, pathology, teeth

## Abstract

Dermatology encompasses the evaluation and management of localized conditions that affect the nail plate, including trauma, primary dermatoses involving the nail bed and nail matrix, and systemic disorders with manifestations that impact the nail unit. In addition to traumatic events that can alter the nail unit, localized conditions such as periungual dermatitis and infections, as well as generalized diseases, can affect the nail matrix, resulting in temporary growth arrest of the nail plate. This can present as a groove (Beau’s line) in the nail plate that progressively grows out as the nail continues to grow. Forensic pathology includes forensic anthropology. Disease and starvation are the most common etiologies associated with growth arrest of the long bones near the epiphyseal plate; the radiolucent horizontal bands (Harris lines) are only observable on the roentgenogram. Localized injuries, malnutrition, and childhood illnesses can result in growth arrest of enamel formation in the teeth (linear enamel hypoplasia); this condition appears as white pits, grooves, and/or bands on the crowns of permanent teeth. A 65-year-old man experienced a trauma-associated Beau’s line on his left great toenail plate. He developed a subungual hematoma; approximately 10 weeks after the injury, he noted the appearance of a Beau’s line. Growth arrest of the nail plate (Beau’s lines), growth arrest of the long bones near the epiphyseal plate (Harris lines), and growth arrest of the teeth’s enamel formation (linear enamel hypoplasia) can be caused by adverse events such as systemic illnesses, malnutrition, medication effects, and trauma. In conclusion, diagnostic stigmata of growth arrest, such as Beau’s lines of the nail plate, Harris lines of the long bones, and linear enamel hypoplasia of the teeth, can be detected during the cutaneous inspection of the nails, the radiologic evaluation of the long bone, and the oral examination of the teeth.

## Introduction

Dermatology is the study of skin, hair, and nails. Changes observed may reflect localized conditions or systemic disorders. A horizontal line across the nail plate (Beau’s line) is an indication of a temporary growth arrest of the nail plate [1-7].

Forensic pathology encompasses several subspecialties, including ballistics, entomology, odontology, osteology, and anthropology. Forensic anthropology involves the evaluation of skeletal remains of decedents to provide a biological profile, which may include information regarding the age, sex, stature, and ancestry of the individual. Distinctive features indicative of periods of growth arrest may be observed on the long bones (Harris lines) and teeth (linear enamel hypoplasia) [8-17].

A 65-year-old man who experienced a traumatic injury to the proximal nail fold of his left great toenail is described. The injury was painful, and within two weeks, he developed a subungual hematoma that presented as black discoloration of the proximal nail fold and the proximal nail. A Beau’s line appeared, within three months, as a transverse groove at the proximal nail fold and progressively moved distally as the nail plate grew. The dermatology-related features of temporary growth arrest affecting the nail plate (Beau’s line) of the reported patient are presented, and the forensic pathology-associated features affecting the epiphyseal region of the long bones (Harris lines) and the teeth (linear enamel hypoplasia) are summarized.

## Case presentation

A 65-year-old man traumatically injured his distal left great toe when he accidentally dropped a heavy plastic container from a 3-foot height onto the proximal nail fold of his toe. The acute pain was severe, and the distal digit remained tender for nearly a day. The left proximal toenail fold developed a black discoloration (pseudo-Hutchinson sign), and the proximal nail appeared black within two weeks following the incident. During the subsequent weeks, the black discoloration of the subungual hematoma continued to expand to a maximum length of nine millimeters and a width of 15 mm. The normal-appearing nail plate of 3-4 mm was noted proximal to the subungual hematoma 18 weeks after the injury had occurred, which confirmed the diagnosis of pseudo-Hutchinson sign; the details and images of this patient’s subungual hematoma and transient proximal nailfold discoloration have previously been published [18].

**Figure 1:**
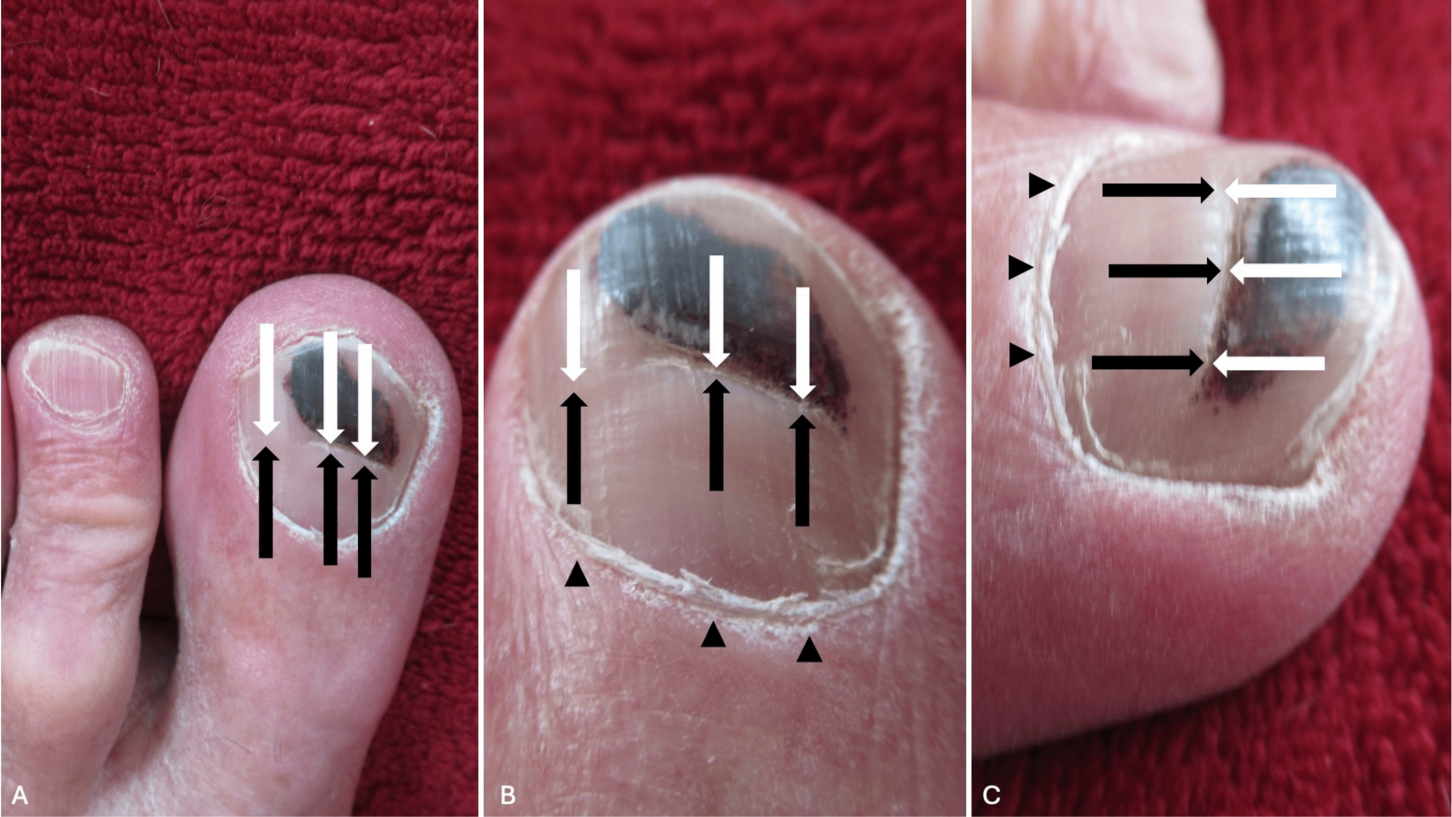
Beau’s line on the left great toenail of a 65-year-old man Distant top view (A), closer top view (B), and closer lateral view (C) of the left great toe. Beau’s line appears as the horizontal groove (between the black and the white arrows) and extends transversely across the nail plate from the lateral nailfolds on each side of the toe. The trauma to the proximal nail fold, when a plastic container dropped onto the proximal nailfold (black triangles), was the etiology of Beau’s line; the appearance of the subungual hematoma (black discoloration of the distal nail plate) occurred prior to the presentation of Beau’s line.

A horizontal groove (Beau’s line) appeared distal to the proximal nailfold approximately 10 weeks after the injury occurred. As the nail continued to grow, the nail plate proximal to the linear groove was thickened. Examination 9½ months after the accident occurred showed that the transverse groove on the left great toenail plate was 9 mm distal to the left great toe proximal nailfold (Figure 1). The Beau’s line continues to move toward the free end of the nail plate on follow-up evaluations.

## Discussion

The nail plate can be an indication of systemic disease or localized conditions. Koilonychia (spoon-shaped nails) can be an indication of iron-deficiency anemia, and Hutchinson sign (black pigmentation of the proximal nail fold) associated with longitudinal melanonychia striatum (a black band extending from the proximal nail fold toward the free edge of the nail plate) can be the clinical stigmata of a subungual malignant melanoma. Beau’s line (a transverse depression of the nail plate extending from the lateral nail fold on one side of the nail to the opposite side) can affect either a single nail as the result of injury to the nail matrix and temporary cessation of nail plate growth or multiple nails when it occurs as the result of a generalized condition (e.g., severe systemic disease) occurring in the individual and causing simultaneous growth arrest of more than one nail [1-5].

The evolution of transverse grooves in the nail plate was commented upon in an article that was published in 1846. The author was a French cardiologist from Paris, Joseph Honoré Siméon Beau (1806-1865). Beau described the appearance of the horizontal nail plate grooves in patients who had typhoid fever; indeed, he postulated that the duration of the disease was proportional to the width of the furrow or depression [3,6].

Beau’s lines occurring on multiple nails have been observed, which is associated with several conditions, including chemotherapy administration, diabetes mellitus, drug ingestion (e.g., antibiotics, antiepileptic agents, and retinoids) and medication allergies, dysmenorrhea, febrile episodes with high fevers, infections (e.g., erythema nodosum leprosum, hand-foot-mouth disease, and syphilis), Kawasaki’s disease, intrauterine stress, myocardial infarction, nutritional deficiencies, renal failure, surgery, and surgical anesthesia. One or more individual nails may be affected by Beau’s line in an individual who has an adverse event occurring on the distal aspect of a specific digit, such as periungual dermatitis, psoriasis, or trauma (Table 1) [1-17]. The patient described experiencing a traumatic event that only involved his left great toenail.

**Table 1:** Comparison of features of Beau’s lines, Harris lines, and linear enamel hypoplasia [1-17]

Feature	Beau’s lines	Harris lines	Linear enamel hypoplasia
Location	One or more fingernails and/or toenails	Epiphyseal end of long bones	Permanent teeth
Etiology	Multiple nails: chemotherapy administration, diabetes mellitus, drug ingestion and medication allergies (antibiotics, antiepileptic agents, and retinoids), dysmenorrhea, febrile episodes with high fevers, infections (erythema nodosum leprosum, hand-foot-mouth disease, and syphilis), Kawasaki’s disease, intrauterine stress, myocardial infarction, nutritional deficiencies, renal failure, surgery, and surgical anesthesia Single nails: periungual dermatitis, psoriasis, and trauma	Disease and diet. Anthropological (deprivation of living conditions, famine, illness and plague), endocrinologic (Cushing syndrome, diabetes, and psychosocial short stature), medication (bisphosphonates, chemotherapy, and immunotherapy), metabolic (ethanol, heavy metal intoxication, and vitamin abnormalities), posttraumatic (nonaccidental trauma and Salter-Harris fracture), physiologic (saltation and stasis), and systemic (chronic anemia, infection, and hematologic neoplasm)	Childhood illnesses, injuries, and malnutrition. In addition, children with exposure to cigarette smoking and low socioeconomic status were at increased risk of developing enamel hypoplasia. The nutritional deficiencies include minerals, proteins, and vitamins. Also, infections (measles, otitis, respiratory, rubella, syphilis, tetanus, urinary tract, and varicella) and metabolic disorders (asthma, diabetes, and diseases of the following glands: adrenal, parathyroid, pituitary, and thyroid)
Age estimation when the injury occurred	Appears one to three months after a finger injury or two to six months after a toe injury	Several methods have been used to estimate the age, with significant intra-observer and interobserver variability	The methods are imprecise and do not conform to the current concepts of the enamel formation process
Symptoms	None	None	Carious teeth and their sequelae, inability to chew food, repeated or failing restorations, and sensitivity to air, cold, warm, and mechanical stimuli
Morphologic presentation	Transverse groove across nail plate forms lateral nail fold on one side to opposite side of digit; may be associated with proximal shedding of the nail plate	Linear radiolucent line (of increased bone density) at the distal epiphyseal area of a long bone visualized on the roentgenogram of the affected long bone of the arm and/or leg; the lines temporally correspond to the period when the etiology is active	Single or multiple pits, grooves, and/or white bands around the circumference of the tooth’s crown that are approximately parallel to the cementoenamel junction and perpendicular to the tooth’s long axis; the lines correspond to periods of active etiology
Treatment	Remove the causative etiology and stress-related event. Allow trauma to heal; thereafter, a normal nail plate will continue to grow distally from the nail matrix beneath the proximal nail fold	Resolve causative etiology; restore adequate nutrition in a person with malnutrition	Early diagnosis and risk recognition, adequate nutrition and good oral hygiene, attrition prevention, anticipation of caries and post eruption breakdown, desensitization, maintenance of dental care, mineralization of teeth, pit and fissure sealants, restorations, and appropriate extractions
Sequalae	Once the causative etiology resolves, a normal nail plate grows proximal to Beau’s line, and Beau’s line is no longer present once the nail plate has completely regrown	Some of the lines (which have been formed) may be removed by subsequent bone remodeling	Malocclusion, poor esthetics, predisposition to dental caries, and tooth sensitivity
Pathogenesis	Growth arrest of the nail plate because of the temporary cessation of nail matrix activity	Growth arrest of long bones near the epiphyseal plate	Growth arrest of tooth enamel formation

Fingernails and toenails grow at different rates. Nail growth on the fingers is typically three times faster than on the toes. Therefore, fingernails grow approximately three millimeters per month, and toenails grow one millimeter per month. In general, Beau’s lines are recognized four to eight weeks after the associated incident on the fingernails and three to even six months after the causative etiology on the toenails [3,6]. Beau’s line on the patient’s left great toenail was initially noticed about 10 weeks after the associated traumatic incident.

Beau’s lines can be associated with transverse leukonychia; this is a horizontal white band that is also present in the nail plate groove [6]. If the etiology was traumatic and associated with bleeding beneath the nail plate, an associated subungual hematoma may precede the appearance of Beau’s line, as in the man described in this report [6]. If the duration of the adverse event is prolonged, complete discontinuation of nail plate growth can result in proximal shedding of the nail plate (onychomadesis) [3,6,7].

Beau’s lines result from nail matrix growth arrest. However, the mechanism of pathogenesis that results in the retardation of normal nail growth when it occurs in the setting of drug exposure, fever, infection, and systemic disease remains to be determined. Four, not necessarily mutually exclusive, etiologies have been speculated [3].

The first etiology is that cellular proliferation may be inhibited; this would be a plausible explanation for Beau’s lines that occur when patients are receiving chemotherapeutic drugs with antimitotic activity. A second possibility is that the quality of the nail plate is altered (such that the nail becomes thinner and dystrophic); however, during this alteration, the activity of the nail matrix and growth rate of the nail plate remain intact. A third alternative is that nail injury or dysfunction causes nail growth retardation; this could occur in the setting of median nerve damage, reflex sympathetic dystrophy in children, and trauma to the hand. Finally, as in the reported patient, direct damage to the nail matrix resulting from trauma or inflammation that affects the periungual and/or matrix regions of the distal digit can lead to a horizontal groove or proximal shedding of the nail [3].

**Figure 2:**
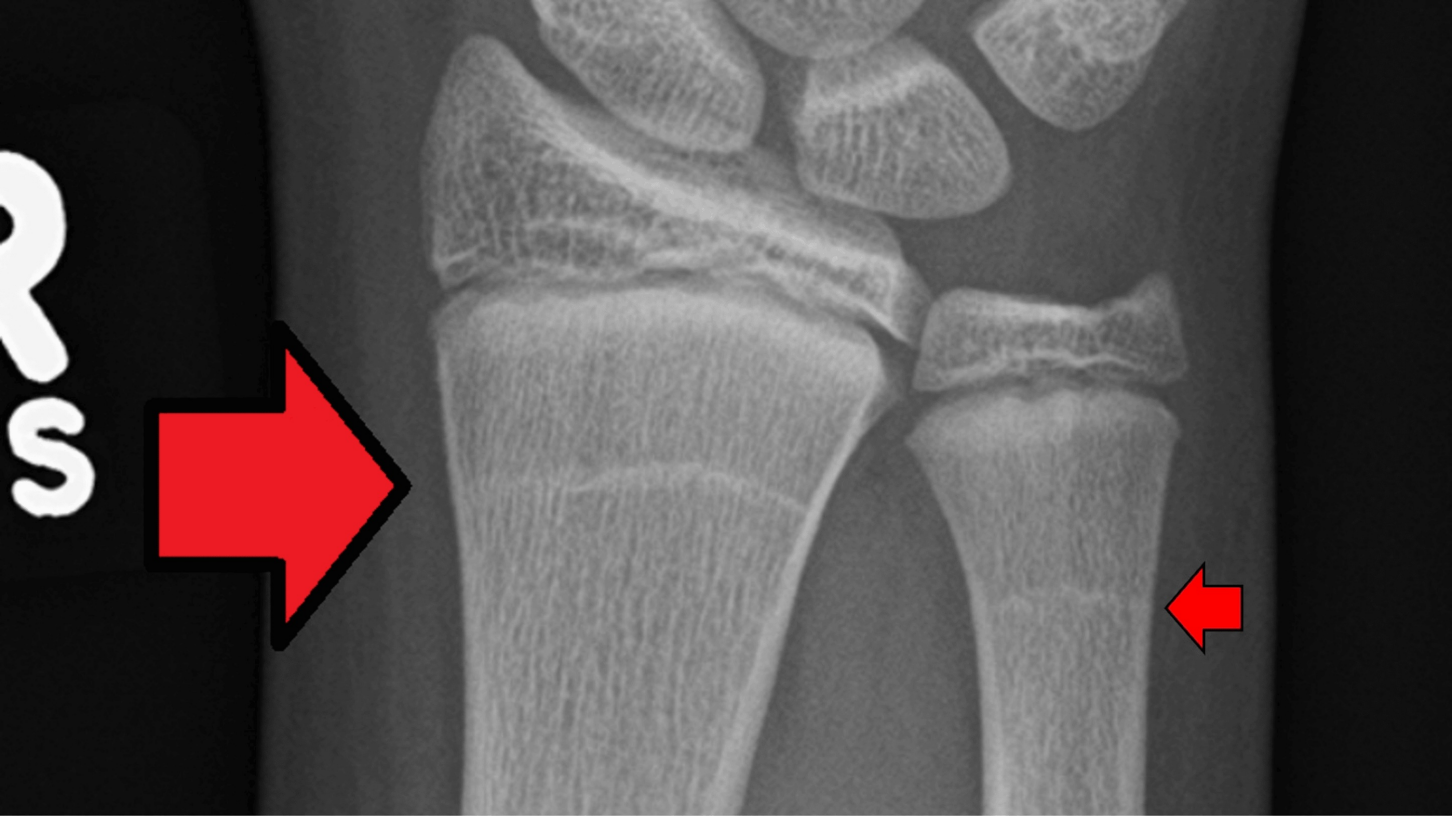
Harris lines on the distal radius and ulna Radiopaque Harris growth arrest lines are present on the right distal radius and ulna (demonstrated by orange arrows with black outlines) in a 13-year-old boy with an underlying bone disease. The photograph was provided on April 13, 2017, by James Heilman, MD. Permission for free republishing is granted; the file is licensed under the Creative Commons Attribution-Share Alike 4.0 International license.

Harris lines are transverse sclerotic radiopaque lines within the metaphases and proximal diaphysis of long bones. They are most often noticed in the distal femur and the proximal tibia; however, they can also occur in the distal portions of the long bones of the upper extremity (Figure 2). They represent a temporary arrest of the longitudinal growth of the long bone [8-12].

Herbert Albert Harris (1886-1968) was a professor of anatomy at the University of Cambridge. In 1926, he reported his research on diabetic children, including the causative factors and the formation of transverse lines in their long bones. In 1927, Edwards A. Parks (1877-1969), a pediatrician, co-authored a paper describing similar observations. Similar lines were observed earlier by Wegner (1874) in the setting of rickets, by Ludloff (1903) from physiologic development around the knees, and by Stettner in healthy and sick children (1920 and 1921). Although the long bone growth arrest lines are occasionally referred to as Park-Harris lines, they are most commonly designated as Harris lines [8-12].

Harris lines have historically been attributed to juvenile malnutrition (starvation), diseases, and psychogenic stress. However, Harris lines can be associated with endocrinopathies, medications, metabolic derangements, systemic illnesses, and trauma (Table 1) [1-17]. The lines are transverse bands of increased radiodensity (which histologically consist of zones of increased thickness of trabecular bone) that can be observed on plain radiographs, computerized axial tomography scans, magnetic resonance images, or positron-emitting tomography bone scans (by increased uptake of 18-fluorodeoxyglucose) [8-12].

Investigators claim to be able to determine the age of the patient or the decedent when the etiologic event caused the Harris lines. However, the evaluation of Harris lines is characterized not only by significant intra-observer variability and high inter-observer biases but also by at least three different methods to determine age. The lines can be noted as early as six weeks following an injury. However, as the individual ages, dynamic bone remodeling occurs, and the previously formed Harris lines may disappear [8-12].

Several mechanisms of the pathogenesis of Harris lines have been postulated. Longitudinal growth arrest of the long bone is a required component in the formation of the transverse line; however, some investigators propose that the appearance of the lines is caused by the recovery of the bone and its regrowth. Associated etiologies that may influence the pathogenesis of the retardation of bone growth include childhood illnesses, hematologic neoplasms, malnutrition syndromes, medications (such as bisphosphonate, immunotherapy, isotretinoin, methotrexate, and other systemic chemotherapy), metabolic disorders (such as endocrinopathies), prolonged immobility, and trauma [8-12].

**Figure 3:**
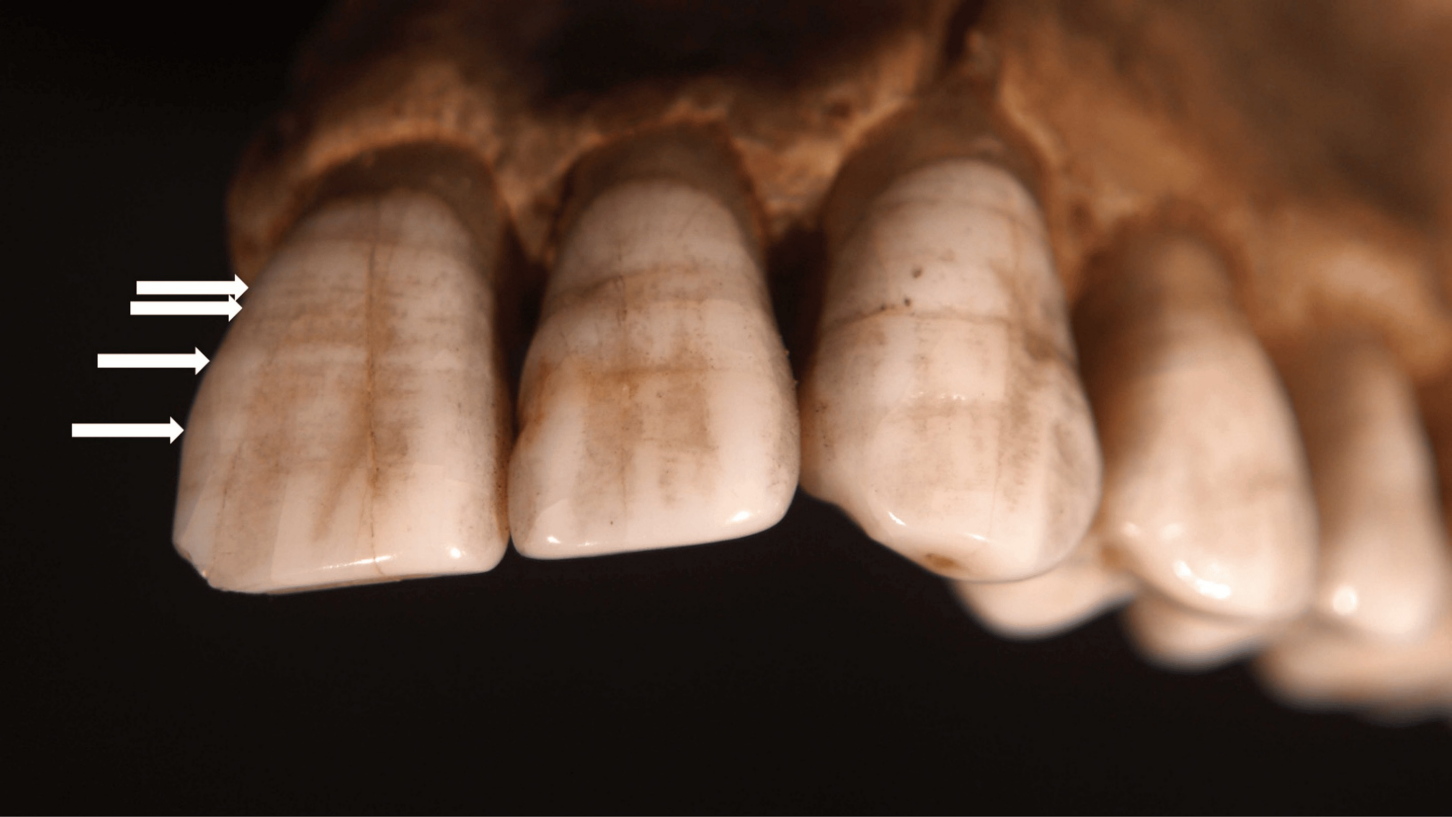
Linear enamel hypoplasia The teeth show white-appearing horizontal lines of linear enamel hypoplasia (demonstrated by the white arrows) on their surfaces. The enamel hypoplasia lines demonstrate the defects of enamel that form during crown development because of either nutritional stress or disease during infancy and childhood. The photograph was provided by Brian Spatola on October 22, 2007, from the Otis Historical Archives National Museum of Health & Medicine. Permission for free republishing is granted; the file is licensed under the Creative Commons Attribution 2.0 Generic license.

Linear enamel hypoplasia is an environmentally associated hypoplasia of the permanent teeth [13-17]. It typically affects multiple teeth; one or more symmetric white bands develop around the circumference of the tooth’s crown (Figure 3). The bands are perpendicular to the tooth’s long axis and approximately parallel to the cementoenamel junction. Each band corresponds to an episode of the causative etiology (Table 1) [1-17].

In contrast to Beau’s lines and Harris lines, linear enamel hypoplasia may be symptomatic. Affected individuals may experience sensitivity to air, cold, warmth, and mechanical stimuli. In addition, some individuals with linear enamel hypoplasia experience carious teeth, difficulty chewing food, and repeated or failing restorations [13-17].

Linear enamel hypoplasia occurs when there is a temporary interruption of the matrix secretion by the ameloblasts. Ameloblasts are the enamel-forming cells; they begin secreting enamel shortly after the dentin-forming cells (odontoblasts) lay down a dentin matrix platform. The enamel is laid down in a series of layers, beginning with the cuspal enamel and subsequently the lateral enamel of the tooth’s crown. Stressful events to the individual are observed as defects in the enamel of the crown (such as pits, grooves, or lines of enamel loss) when they occur during the lateral enamel formation [13-17].

Environmental stresses associated with linear enamel hypoplasia predominantly include childhood illnesses, injuries, and malnutrition. Children from low socioeconomic status and who have been exposed to cigarette smoking are at increased risk of developing enamel hypoplasia. Several other factors that have been associated with linear enamel hypoplasia include infections (such as measles, otitis, respiratory infections, rubella, syphilis, tetanus, urinary tract infections, and varicella), metabolic disorders (such as asthma, diabetes, and diseases of the following glands: adrenal, parathyroid, pituitary, and thyroid), mineral deficiencies (such as calcium, fluorine, magnesium, and phosphorus), protein deficiencies (such as amelogenin, enamelin, and tuftelin), and vitamin deficiencies (such as vitamins A, C, D, and K) [13-17].

The treatment of linear enamel hypoplasia is the prevention of future episodes and monitoring for adverse sequelae. Good oral hygiene and providing adequate nutrition are of paramount importance. In addition to early diagnosis and risk recognition, management of linear enamel hypoplasia includes maintenance of dental care (including anticipation of caries and post-eruption breakdown), desensitization, prevention of attrition (by using acrylic jigs and custom-made bite blocks), mineralization (with fluoride and calcium phosphate agents), sealants for pits and fissures, restorations (including glass ionomer cement, composite, stainless steel crowns, full veneer metal-ceramic crowns, fixed-removable partial dentures, and implants), and extractions (if the teeth are non-restorable). Malocclusion, poor esthetics, predisposition to dental caries, and tooth sensitivity are potential adverse outcomes for individuals who develop linear enamel hypoplasia [13-17].

## Conclusions

Growth arrest of nail plates can present as Beau’s lines, of long bones can appear as Harris lines, and of enamel formation of teeth can result in the development of linear enamel hypoplasia. Identification of the stigmata of growth arrest in a patient or a decedent can provide insight into the occurrence of a prior adverse event, such as systemic illness, malnutrition, trauma, or the effects of medications. The case report of a dermatology-related presentation of growth arrest in a 65-year-old man with a subungual hematoma associated with trauma to the proximal nail fold of his left great toe was described, and a narrative review of forensic pathology-associated manifestations of growth arrest was presented. In conclusion, morphologic clues to localized trauma or generalized disease in an individual can be observed by the astute clinician during the evaluation of the nails, long bones, or teeth.
